# The economics of ethnic marriages: Endogamy and the social status of minority groups

**DOI:** 10.1111/1468-4446.13133

**Published:** 2024-07-14

**Authors:** Naim Bro, Liran Morav

**Affiliations:** ^1^ Adolfo Ibañez University Penalolen Chile; ^2^ Millennium Institute Foundational Research on Data Santiago Chile; ^3^ University of Cambridge Cambridge UK

**Keywords:** Chile, ethnic endogamy, Jewish, Mapuche, socioeconomic status

## Abstract

This study examines the relationship between ethnic endogamy and socioeconomic status (SES) within the socioeconomically divergent Jewish and Native‐Chilean Mapuche communities of Santiago, Chile. By leveraging the Hispanic naming convention to analyze dual ethnic surnames, we trace endogamy patterns across comprehensive datasets that go back to 1884 up to the present. Our quantile regression analysis reveals that individuals from the lower SES brackets of the Jewish community and the higher brackets of the Mapuche community are more likely to have mixed ethnic backgrounds. This finding shows a nuanced interplay between socioeconomic standing and marital choices, suggesting that these factors significantly influence the persistence and transformation of SES within minority groups. The study introduces the Ecological Model of Ethnic Disaffiliation, providing a theoretical framework that explains how socioeconomic outliers within ethnic groups could lead to a narrowing of their socioeconomic range over generations.

## 1 INTRODUCTION

This article delves into the interplay between socioeconomic status and marital choices within minority communities, focusing on the Jewish and Mapuche populations in Santiago, Chile. At its core, this study reveals a compelling dynamic: individuals who significantly deviate from the predominant socioeconomic status of their ethnic group are more inclined towards mixed‐ethnic marriages. Conversely, those marrying within their ethnic group often share a similar economic standing with their peers. This phenomenon, which we term the “Ecological Model of Ethnic Disaffiliation” (EMED), suggests that the socioeconomic niche of minority groups is perpetuated over time as members with atypical socioeconomic profiles are more likely to exit these communities and/or more likely to have mixed‐ethnic children who are themselves more likely to exit the same.

Central to our analysis is the contrasting socioeconomic profiles of the Jewish and Mapuche communities in Santiago. The Jewish community, with an organizational genesis in the early 20th century (Navarro Rosenblatt, 2018; Severey, 1956), experienced a significant influx of members during World War II (refer to Figure 7). Associated to upward mobility (Stern, 2017), this community presents a stark contrast to the Mapuche, Chile's largest indigenous group. The Mapuche people, primarily originating from southern Chile, have undergone a massive urban migration, with about half settling in Santiago in the last few decades (Imilán & Álvarez, 2008). In this urban setting, they generally occupy lower socio‐economic positions (Agostini et al., 2008; Antón & Carrera, 2011). The socioeconomic divergence between the two communities^1^ offers a compelling perspective for examining the interplay of endogamy and socioeconomic status (SES).

Employing a novel methodology that leverages the Hispanic naming convention, we trace endogamous unions and their correlation with SES. This approach involves identifying individuals with two Jewish or Mapuche last names and analyzing comprehensive datasets ranging from 1884 to the present. The quantile regression analysis uncovers that lower SES brackets within the Jewish community and higher brackets within the Mapuche community are more prone to mixed ethnic marriages. This finding highlights the nuanced interaction between SES and marital choices, suggesting significant implications for the persistence and transformation of SES within these minority groups.

Chile's socio‐economic structure, characterized by significant inequalities (UNDP, 2017), makes it an ideal context for exploring these dynamics. The study goes beyond traditional analyses of ethnic minority economics, offering new insights into how socioeconomic factors and marital decisions within ethnic groups intersect and evolve. The subsequent sections will further dissect theories related to the socioeconomic outcomes of ethnic minorities, detail our data sources and empirical strategy, and provide an in‐depth analysis of the Jewish and Mapuche groups in Santiago. Our findings not only illuminate the relationship between endogamy and SES but also advance our understanding of the processes driving socioeconomic differentiation within ethnic minorities. We conclude with reflections on the broader implications of our study, suggesting pathways for future research and policy development, and contributing a novel perspective to the discourse on the economics of ethnic marriages.

## 2 THE SOCIOECONOMIC OUTCOMES OF ETHNIC MINORITIES

Ethnic affiliation is a significant predictor of an individual's socioeconomic status in many country contexts, with various theoretical models explaining the persistent socioeconomic advantages or disadvantages of specific ethnic minority groups. These can be classified into three categories: human capital models, which highlight the socio‐economic impacts of ethnically‐preponderant skills and behaviors; social capital models, where the emphasis is on the effects of ethnically‐patterned relational networks and institutions; and macro‐structural explanations, which focus on the ways that broader societal institutions systematically advantage some groups while disadvantaging others.

Human capital theories highlight the role of education, skills, and economically productive cultural values and behaviors specific to an ethnic minority. A classic contribution in this field is Borjas' (1992) economic study of what he conceptualised as US ethnic minorities' “ethnic capital,” defined as the average human capital of the ethnic environment in which an individual ethnic minority member is socialised. Borjas showed that the skills of a range of second‐generation ethnic minorities depended not just on parental investments in their children's skills and education, but also on the ethnic capital of children's ethnic communities. Human capital explanations are very common to explain the economic success of Jewish communities around the world (Burstein, 2007). An example of economically advantageous values is Jews' two millennia‐old religious obligation to acquire literacy (Botticini & Eckstein, 2012), or the tendency of certain ethnic groups to gravitate towards particular professions (e.g., for British South Asians see Modood & Khattab, 2016; for Asian Americans see Lee & Zhou, 2016).

A similar narrative is presented in Jewish contexts. Tunisian Jews migrating to France in the 1950–1970s, for example, gravitated towards medical studies, thereby integrating into France's middle classes strata (Bensimon, 1970). Conversely, Tunisian Muslims arriving in France during the same period were frequently circular or seasonal migrants. They therefore opted for flexible low‐skill jobs and eschewed investments in education. Such differences in educational and settlement orientation help explain why a significantly larger share of first‐generation Tunisian‐Jewish migrants achieved a middle‐class social status in France compared to Tunisian Muslims (Bruno, 2010). Furthermore, much like Lee and Zhou's account of Asian‐American scholastic over‐achievement, Fejgin's (1995) statistical analysis finds that Jewish‐American youths' favorable school performance compared to other minorities is mediated by parental educational expectations.

The second category of explanations is *social capital*, focused on the capacity of individuals to mobilise resources in interpersonal and ethnic‐community networks (Lin, 2001). This concept helps explain the success of ethnic businesses, such as Asian‐ and Cuban‐Americans, which benefit from the mobilization of family labor and co‐ethnic support (Light, 1984; Portes & Manning, 1986; Sanders & Nee, 2014). Ethnic institutions play a crucial role in reinforcing ethnic capital within communities. In Jewish contexts across Europe and North America, ethnic welfare organizations have significantly contributed to poverty reduction (Liedtke, 1999; Wolfe, 1974; Zytnicki, 2003). In Santiago, Jewish welfare organizations established as early as 1917 have supported disadvantaged community members (Tapia‐Adler, 2013). Additionally, credit associations and loan societies have been instrumental in providing business credit, aiding numerous Jewish migrants in establishing businesses and securing housing, especially in the US, Britain, and France (Fourtage, 2019; Light, 1984; Moya, 2005; Tananbaum, 2015).

The effectiveness of social capital relies heavily on trust and reciprocity within ethnic communities, as demonstrated by Richman's (2006) study of New York's Jewish diamond traders. Their interconnected networks facilitate access to valuable information and ensure adherence to informal trade obligations, underpinning their economic success.

Social capital models, like human capital ones, focus on minority groups' self‐driven economic mobility, but they operate within broader macro‐political and economic contexts that often limit their opportunities. *Macro‐structural explanations*, pivotal in critical sociology, delve into societal inequalities and systemic disadvantages impacting racialized minorities (Bonilla‐Silva, 2006; Meghji, 2021). These macro‐structural mechanisms include ideologies, economic policies, and political structures that perpetuate inequalities across various institutional domains, resulting in what is termed as “ethnic penalties” (Khattab & Johnston, 2015; Modood & Khattab, 2016).

In Chile, these macro‐structural factors have profoundly affected the Mapuche. A history of post‐colonial land seizures and assimilation policies has marginalized the Mapuche, leading to significant poverty and illiteracy compared to the non‐indigenous population (Alberti et al., 2023; Valenzuela & Unzueta, 2015). Ethnic stratification, a key component of these macro‐structural processes, restricts minorities' access to resources and opportunities by enforcing interethnic boundaries. In the US, historical examples include anti‐black segregation laws and quotas against Jews in elite universities (Alba, 2008; Patterson, 2005; Perlmann & Waldinger, 1997). In Chile, the stratification in schools since the 1980s has exacerbated social inequalities (Carnoy, 2000).

The model presented in this paper is not at odds with the more common explanations outlined above but functions as a complementary mechanism that consolidates the economic niche position of ethnic minorities over time. The next section outlines the EMED model.

## 3 THE EMED MODEL OF ETHNIC MARRIAGE AND THE CONSOLIDATION OF GROUP STATUS

This paper presents the 'Ecological Model of Ethnic Disaffiliation' (EMED), a theoretical framework explaining the persistence of economic outcomes within ethnic minorities through exogamous marriages. It posits that an individual's economic status influences their likelihood of marrying outside their ethnic group, which consolidates the group's economic position by integrating or excluding economically atypical members or their mixed‐ethnic children.

EMED adapts McPherson's (1983) ecological model of organizational affiliation, suggesting that members most likely to leave an organization are those at the periphery of the organization's social space. We apply this concept to ethnic groups, where network homophily leads to socio‐demographically atypical members marrying outside their ethnic group and possibly disassociating from their ethnic category.

The model's empirical assumptions are:

(1) Ethnic minorities' social ties sociability sites influence marital choices;
(2) Socioeconomically atypical members have limited co‐ethnic interaction opportunities, increasing exogamous marriage likelihood;
(3) Such marriages promote ethnic disidentification.

Let us discuss these assumptions in turn.

Research indicates that the spousal choices of ethnic minorities are influenced by their socialization environments, including social clubs, educational institutions, and neighborhood networks (Eve, 2010; Kalmijn, 1998; Kalmijn et al., 2006; Verbrugge, 1977). Families play a crucial role in encouraging their children to engage with these spaces, as demonstrated by Kalmijn and Flap (2001) in the case of Jewish families in the Netherlands. Structural factors, rather than personal attractions, predominantly determine spousal choice, particularly in communities that establish ethnically‐focused social institutions to encourage endogamy (O’Leary & Finnäs, 2002).

Marital homogamy is often influenced by shared educational and socioeconomic backgrounds, with individuals likely marrying those from similar social circles and environments (Blackwell & Lichter, 2004; Kalmijn, 1998). However, socioeconomically atypical individuals within their ethnic groups find fewer opportunities for such interactions, which might lead to outmarriage (Kulczycki & Lobo, 2019; Okun & Khait‐Marelly, 2010). For the Mapuche case, Valenzuela and Unzueta (2015) state that “having non‐indigenous partner is a selective phenomenon for Mapuche that increases with urban residence and educational level” (p. 2093).

Finally, the third assumption is that intergenerational ethnic transmission is challenging among mixed‐ethnic children, who often disidentify from their minority ethnic group. This phenomenon has been noted in Jewish communities across Europe and North America (DellaPergola, 2016); for instance, Phillips (2018) observes that Jewish‐gentile couples in Chicago are less likely to reside in Jewish neighborhoods compared to Jewish‐Jewish couples. For Chile, Mateo & Valenzuela (2016) show the importance of having Mapuche last names for the subjective ethnic classification of Mapuches, an attribute that probabilistically less frequent among mixed couples. Likewise, Brablec (2021) describes the challenges of persons of Mapuche/non‐Mapuche parents in Santiago to maintain the Mapuche identity.

(There is a related but not identical literature which shows that socioeconomically atypical group member disidentify ethnically more than socioeconomically typical group members in the United States (e.g., Saperstein & Penner, 2012) and Brazil (e.g., Telles, 2004). For Chile, Valenzuela and Unzueta (2015) show that higher income Mapuches do not disidentify more than lower income Mapuches; although qualitative findings by Sepúlveda (2023) indicate that a subset of upwardly mobile Mapuches do experience significant disidentification. Our study does not evaluate the direct impact of status on disidentification, but only through the mediating effect of outmarriage.)

Next, we discuss the data sources used to empirically demonstrate the EMED model using the case of the Jewish and Mapuche communities of Santiago.

## 4 DATA SOURCES

The main dataset used in the analysis comprises the near entirety of Santiago's Jewish and Mapuche residents of Santiago who are registered to vote in political elections. In other words, the present analysis does not generalize from statistical samples of Santiago Jews/Mapuches; instead, we test our empirical hypotheses on a dataset containing the majority of adult Jews/Mapuches in the city. The individuals in each group were identified on the basis of their surnames. The data also include information on the socioeconomic, geographic and family characteristics of each Jewish/Mapuche individual. To compile these data, we drew on: (i) lists of *ethnic surnames* used to identify Jewish and Mapuche residents of Santiago; (ii) a *socio‐demographic dataset* of Santiago's population, which was used to compute the socioeconomic characteristics of each of the Jewish/Mapuche individuals in our main samples; (iii) Chile's *historical Civil Registry*, which we used to calculate the familial endogamy characteristics of each Jewish/Mapuche individual's background. More information on each of these datasets is provided below.

### 4.1 Ethnic surnames

Jewish and Mapuche group members tend to have distinctive and rare last names in the Chilean context. For the former, typical last names are Ergas, Rosenberg, Levy, and Cohen. For the latter, Marileo, Collío, Curín, and Mariqueo are common examples. Following Waters' (1990) seminal work on ethnic self‐identification, we assume that surnames are reliable indicators of ethnic identification.

The list of Chilean‐Jewish surnames comes from two sources. The first source is the membership of the *Estadio Israelita*, a recreational center with an exclusively Jewish membership. The list contains 5591 names, each with two surnames (paternal and maternal).^2^ The second source is the Mif'al Ha'haim of 2010, an electoral registry of persons that voted for the leadership of the Jewish community of Chile that year. The list has 912 names and 424 distinct last names. Considering both lists, we took all paternal and maternal last names and removed the ones most common in the wider population. This was a necessary step because not every Jewish person has a distinctively Jewish surname. The procedure to identify a threshold for common names was the following: Levy and Cohen—two common yet distinctively Jewish surnames—are the two most common names among the membership of the Estadio Israelita. In the electoral registry of 2020, these surnames have a frequency of 143 and 180, respectively. Therefore, if a surname of our initial list has a frequency above 180 in the electoral registry, then it was removed from our final list of Jewish surnames. As a result, if an Estadio Israelita member has the surnames Cohen González, only her Cohen surname is included in the final list, because González is a common name in Chile.

**FIGURE 1:**
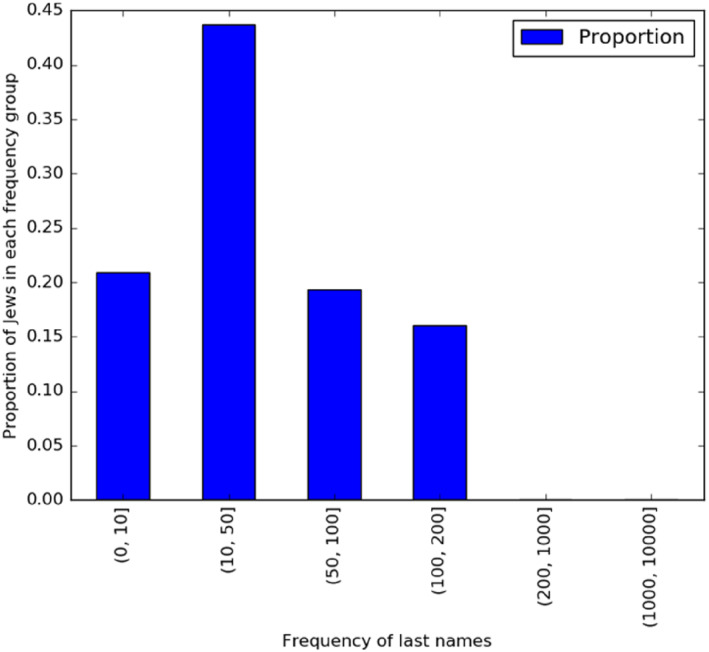
Frequency of Jewish surnames.

To further ensure that we identify Jewish last names correctly, we used the *historical Civil Registry* database (described below) to remove surnames with a Jewish endogamy score below 0.1. The details are explained below, but shortly: if less than 10% of people with a given surname have a Jewish second (first) last name, then it is removed from the final list of Jewish surnames. The consolidated list has 3563 distinct last names, most of them very rare in the wider population. The histogram in Figure 1 shows that a large number of Santiago Jews have surnames held only by 10–50 people.

The list of Mapuche surnames was obtained from compilations made by Amigo and Bustos (2008) and Painemal (2011).^3^ Here, we also removed common names, using the following procedure: we selected all persons with two Mapuche last names, per our initial list, and then looked for the most frequent last names, which were Collío and Marileo. In the wider Santiago population, these last names have a frequency of 686 and 694, respectively, so we used the latter (694) as our threshold to identify common surnames. As we did for Jews, for Mapuches we also removed surnames with an endogamy score below 0.1. Four of the surnames intersected with the Jewish list of surnames, and these were removed from both lists.^4^ The final list contains 8627 surnames.^5^

**FIGURE 2:**
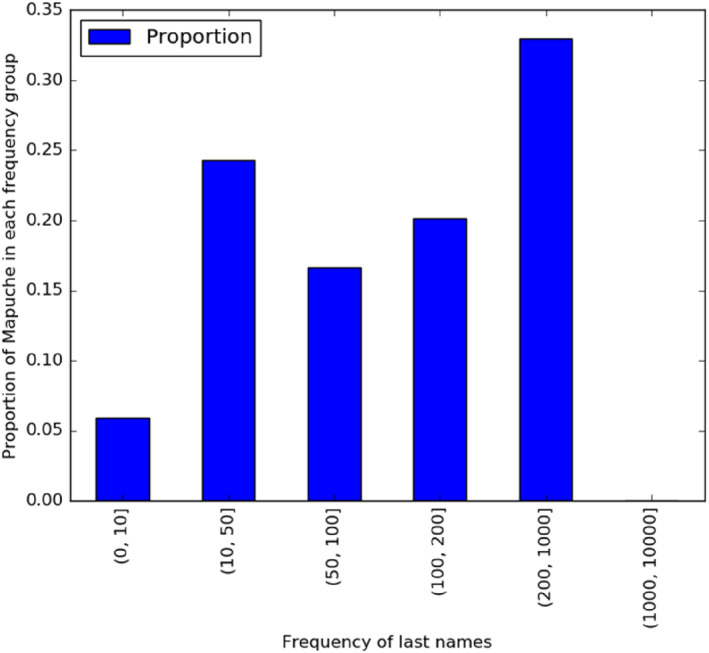
Frequency of Mapuche surnames.

Figure 2 shows the size of the surnames held by Mapuches in Santiago. Less than half hold surnames below a frequency of 100, and about 30% hold surnames in the 200–1000 frequency group. Appendix 1 permits comparing the size of surnames held by Jews and Mapuches and the general population of Santiago. It shows that, in contrast to Jews and Mapuches, typical Santiago residents hold surnames held by more than 1000 individuals.

### 4.2 Socio‐demographic dataset

The data on socio‐demographic characteristics was obtained from Bro and Mendoza (2021); it uses the Chilean electoral registry of 2020, which contains the full name, unique identifying number, and the address of all individuals eligible to vote for political authorities in Chile. Only the 4,652,933 residents of Santiago were included in this analysis. From the electoral registry, we selected the individuals with at least one Jewish or Mapuche last name, per the lists discussed above. We computed the socioeconomic rank of individuals using the Territorial Well‐being Index of 2012 (CIT, 2012), which measures the relative socioeconomic status of every census administrative unit down to the city's 39,901 blocks. From census data, this index aggregates educational and house‐infrastructure features to determine the SES of each urban block.

In this study, we employ socioeconomic status (SES) as a key variable rather than social class. Although social class has proven highly useful in studies of homogamy (e.g., Toft & Jarness, 2021), we chose a simpler metric built from educational and house‐infrastructure indicators aiming to gain in coverage. As mentioned previously, our SES metric covers millions of persons, and provides a very granular metric for positioning individuals within Santiago's social hierarchy.

**FIGURE 3:**
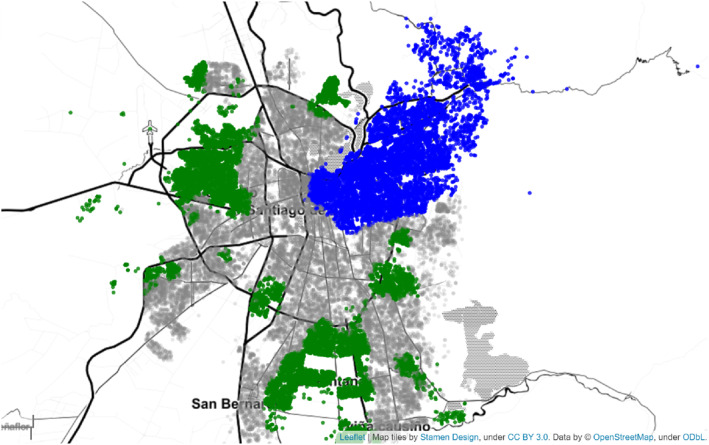
Areas of Santiago with Jewish (blue) and Mapuche (green) overrepresentation. Blue points are areas with Jewish overrepresentation, and green points are areas with Mapuche overrepresentation. This map identifies neighborhoods using the method proposed by Spielman and Logan (2013), where every geocoded unit is represented as concentric circles at increasing distances, and every ring summarises some metric of interest. For any given point, we computed the proportion of Jews/Mapuches among the 200, 700, and 1500 closest neighbors. Then the vectors were classified into three clusters using a Gaussian Mixture Model. The cluster with the highest proportion of Jews was classified as Jewish (blue), the one with the highest proportion of Mapuches was classified as Mapuche (green), and the third cluster was classified as neutral (gray). The visualization was produced using Leaflet for Python.

Figure 3 illustrates the geographic distribution of the Jewish and Mapuche communities in Santiago, with blue points indicating areas of Jewish overrepresentation and green points for Mapuche overrepresentation. The method proposed by Spielman and Logan (2013) is used for neighborhood identification. This visualization helps to understand the spatial dynamics of these communities within Santiago.

### 4.3 Historical civil registry

We use an original dataset that includes all persons registered in the Chilean Civil Registry since its creation in 1884. These are timestamped entries that include the two last names and the registration year of more than 22 million individuals—dead and alive—born since 1884. This dataset allows us to follow the marital trajectories of specific extended families over more than a century.^6^

**FIGURE 4:**
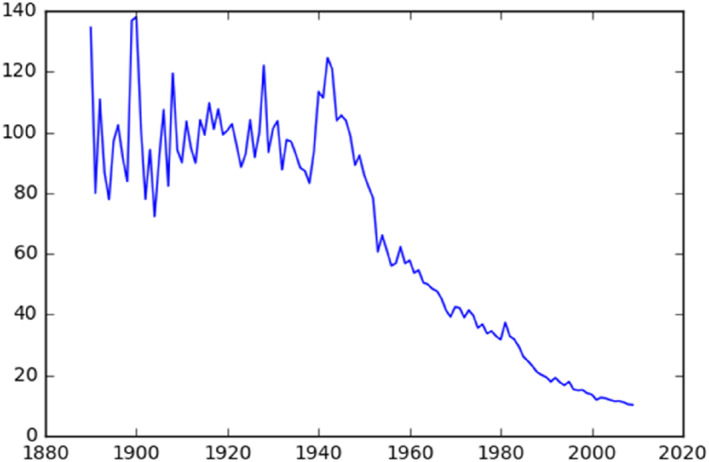
Historical endogamy, Mapuche last names.

**FIGURE 5:**
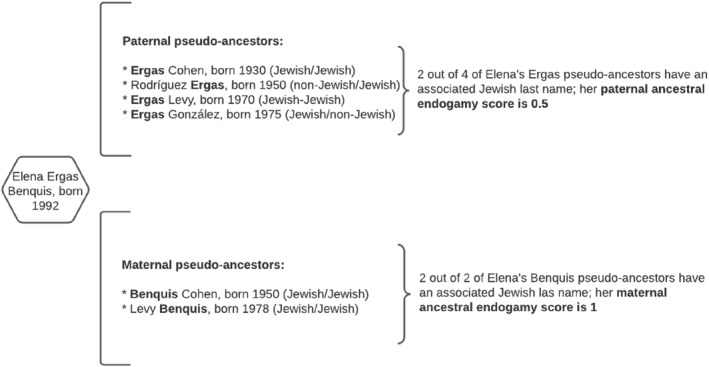
Illustration of how the ancestral endogamy score is calculated.

Figures 4 and 5 outline the changes in endogamy among Jews and Mapuches based on the surname compositions of each group, as found in the historical Civil Registry dataset. The endogamy scores on the *Y* axis are based on Kalmijn's (1998, p. 405) formulas for endogamy odds‐ratios, which takes account of the relative sizes of ethnic groups.^7^

**FIGURE 6:**
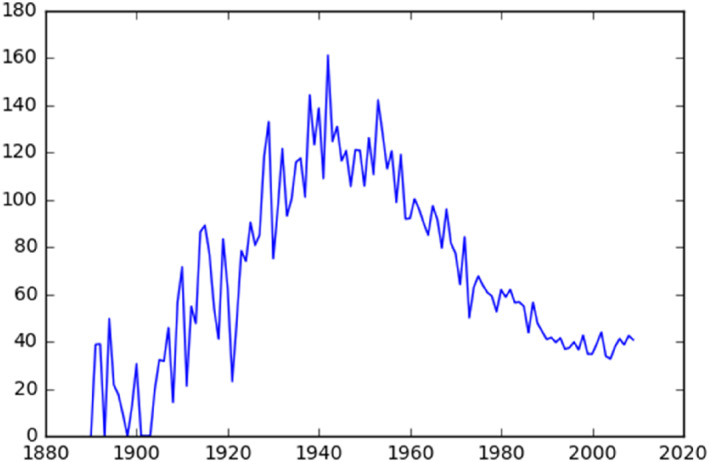
Historical endogamy, Jewish last names.

Figure 6 shows that Jewish endogamy in Chile increased between 1900 and 1940, whereupon it began declining steadily. The low levels of endogamy in the early twentieth century are consistent with historical accounts. A Jewish visitor in Chile in 1935—a few years before endogamy peaked—indicated that “there are many of such marriages, generally between Jewish men and Chilean women. The immediate motive of these ties is probably (…) the lack of a Jewish collective life, especially in the first years [of immigration]” (quoted in Matus, 1993, p. 63).

Endogamy among Mapuches, on the other hand, was volatile around a stable mean between 1900 and roughly 1950, after which it too steadily declined.

## 5 EMPIRICAL STRATEGY

The analysis proceeds in two steps. For each ethnic community, first, we run a quantile regression to estimate the association between parental endogamy and SES, by comparing individuals with two versus one ethnic last names. Then, we run three quantile regressions to examine the link between ancestral endogamy and SES for individuals with two ethnic surnames, a paternal ethnic surname, and a maternal ethnic surname.

### 5.1 Quantile regression

Standard linear regression techniques estimate the relationship between the outcome variable and regressors based on the conditional mean. In contrast, the quantile regression method introduced by Koenker and Basset (1978) allows measuring the impact of the regressors across the entire distribution of the dependent variable. For example, in social mobility research, quantile regression can capture the effect of parental income on their offspring's later‐life income for poor families versus rich families. In this study, quantile regression models are used to estimate if and how the association between ethnic endogamy and relative socioeconomic status vary across the socioeconomic status distribution of Jews/Mapuches in Santiago.

The quantile regression can be expressed as:

$$y_{q}=x_{i}\beta_{q}+e_{i}$$

where *β*_*q*_ is the vector of unknown parameters associated with the *q*th quantile. The function that the quantile regression seeks to minimize can be expressed as:

$$Q\left(\beta_{q}\right)=\sum q\left|y_{i}-x_{i}\beta_{q}\right|+\sum \left(1-q\right)\left|y_{i}-x_{i}\beta_{q}\right|$$

where 0 < *q* < 1.

Since our data is clustered around surnames, we estimate the standard errors using the bootstrapping method for clustered data proposed by Hagemann (2017). We conduct all the analyses using the quantreg package in the R programming language (Koenker, 2022).

The different models have different specifications. Table 1 shows the variables included in each regression. Subsequently, we describe each variable.

**TABLE 1:** Quantile regression models and the variables they include.

Variables	Models
Dependent:	1. Parental endogamy (two vs. one Jewish surname)
SES	
Independent:	
Has two Jewish surnames (dummy variable)	
Controls:	
Age	
Sex	
Paternal surname size (paternal)	
Maternal surname size (maternal)	
Dependent:	2. Ancestral endogamy (among persons with two Jewish surnames)
SES	
Independent:	
Mean endogamy score	
Controls:	
Age	
Sex	
Paternal surname size (paternal)	
Maternal surname size (maternal)	
Family age (mean)	
Dependent:	3. Ancestral endogamy (among persons with paternal Jewish surnames only)
SES	
Independent:	
Paternal endogamy score	
Controls:	
Age	
Sex	
Paternal surname size	
Maternal surname size	
Paternal family age	
Dependent:	4. Ancestral endogamy (among persons with maternal Jewish surnames only)
SES	
Independent:	
Maternal endogamy score	
Controls:	
Age	
Sex	
Paternal surname size	
Maternal surname size	
Maternal family age	
Dependent:	5. Parental endogamy (two vs. one Mapuche surname)
SES	
Independent:	
Has two Mapuche surnames (dummy variable)	
Controls:	
Age	
Sex	
Paternal surname size	
Maternal surname size	
Dependent:	6. Ancestral endogamy (among persons with two Mapuche surnames)
SES	
Independent:	
Mean endogamy score	
Controls:	
Age	
Sex	
Paternal surname size	
Maternal surname size	
Dependent:	7. Ancestral endogamy (among persons with paternal Mapuche surnames)
SES	
Independent:	
Paternal endogamy score	
Controls:	
Age	
Sex	
Paternal surname size	
Maternal surname size	
Dependent:	8. Ancestral endogamy (among persons with maternal Mapuche surnames)
SES	
Independent:	
Maternal endogamy score	
Controls:	
Age	
Sex	
Paternal surname size	
Maternal surname size	

### 5.2 Socioeconomic status

Socioeconomic status, or SES, is a ranked variable used as the dependent variable in all eight regressions. It was computed using the socio‐demographic dataset described above. We used the data compiled by Bro and Mendoza (2021) to link individuals to urban blocks.^8^ Individuals were each assigned a SES rank based on their respective residential blocks in Santiago. Thus, if person A lives in block X, we computed person A's SES as the SES of block X. 80% of Santiago's blocks house less than 218 people, making the imputed SES rank of each individual in the dataset highly granular, given the city's large size.

### 5.3 Parental endogamy

The *parental endogamy* variable is a binary measure that indicates whether an individual is the child of a co‐ethnic or mixed couple. It was computed on the basis of whether the individual has one or two Jewish (or Mapuche) surnames in his or her surname‐pair. We assume that two same‐ethnicity surnames express a higher likelihood of parental endogamy than last names of differing ethnicity. For instance, a Cohen Levy person is considered as being more likely to have *two* Jewish parents than a Cohen González person, whom we will consider more likely to have *one* Jewish parent (both Cohen and Levy are typical Jewish surnames, whereas González is not). The same logic applies in the Mapuche case: we will assume that a, say, Marileo Collío person is more likely to come from an endogamous Mapuche household than a Marileo González person.

### 5.4 Ancestral endogamy

We employ the same principle to construct the *ancestral endogamy* variable. This variable measures the degree of endogamy within an individual's pseudo‐ancestors. We take each person's year of birth, and consult Chile's historical Civil Registry to calculate what proportion of people born before her with the same ethnic surname also have a co‐ethnic associated surname. We used this information to compute an ancestral endogamy score for each ethnic surname associated with an individual in the analytical sample. The average of the two scores represents the individual's ancestral endogamy score. The ancestral endogamy score of individuals with only one ethnic surname is equivalent to that of the single surname.

Figure 5 is a simplified, fictional diagram of how we measure ancestral endogamy. In the example, Elena Ergas Benquis (two Jewish last names) was born in 1992. Four Ergas and two Benquis in the dataset were born before her. These are her pseudo‐ancestors; “pseudo” in the sense that we do not know whether or not each one represents a true ancestor, but they all have a higher‐than‐chance likelihood of belonging to her family tree. Elena's endogamy score is the proportion of pseudo‐ancestors with an associated same‐ethnicity last name, out of the total of pseudo‐ancestors. So, if two out of four of her Ergas pseudo‐ancestors had an associated Jewish last name (meaning that they married endogamously), then Elena's paternal endogamy score is 0.5. Likewise, if two out of two of her Benquis pseudo‐ancestors had an associated Jewish last name, her maternal endogamy score is 1. Notice that the score of a surname changes with year of birth, as the pool of pseudo‐ancestors change. For persons with two ethnic last names such as Elena, the endogamy score is the average of the paternal and the maternal endogamy scores. In the case of persons with one ethnic last name, their endogamy score is the one assigned to their one ethnic last name (paternal or maternal).

### 5.5 Family age

Family age corresponds to the year in which an individual's last name first appeared in the Chilean Civil Registry.

**FIGURE 7:**
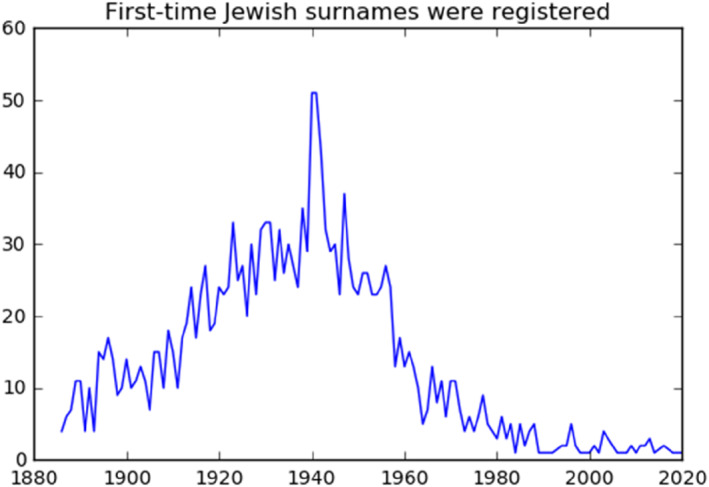
First‐time appearance of Jewish surnames in the civil registry.

**FIGURE 8:**
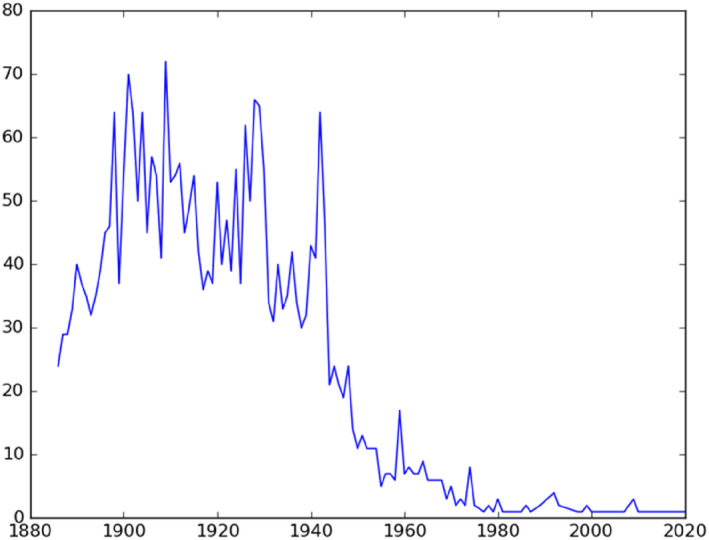
First‐time appearance of Mapuche surnames in the civil registry.

Figures 7 and 8 show when Jewish and Mapuche names were first registered. The two graphs should be read differently. Contrary to Jews, Mapuches were in Chile before the state started collecting their statistics. For this reason, Figure 7 should be interpreted as an index of the penetration of the Chilean state in Mapuche territories. It shows that the Civil Registry kept registering new Mapuche families at high numbers up to the 1940s.

As for Jews—a comparatively recent community in Chile—the figure should be read as an indication of the timing of arrival of Jewish families. Consistently with our knowledge of world historical events, the figure shows that most surnames appear first in the civil registry during World War II, following the exodus of Jews escaping the Holocaust in Europe. We include the “family age” control only in the Jewish regressions, because only for Jews it has a clear conceptual meaning.

### 5.6 Other controls

Another control is *age*. The electoral registry does not include an age variable. However, it does contain each person's national identification number (RUT), which correlates with age. We use a dataset published in Francisco Villena's GitHub repository^9^ to find the slope regressing age from RUT. The intercept of the OLS regression is 1932 and the coefficient of RUT is 3.3e − 06 (the R‐squared is 0.96, reflecting a very good fit). To estimate a person's age, then, we compute the following equation: *AGE*_*i*_ = 1.932*e* + 03 + (*RUT*_*i*_ * 3.337*e* − 06).

Additional controls include s*ex*, which is contained in the Civil Registry Records, and s*urname size*, that is, the number of people in the civil registry with a person's surname.

### 5.7 Filters

To increase the robustness of the results, we removed the following observations from the datasets. First, we removed all ethnic last names with an endogamy score below 0.1. This means that, to be included in the final dataset, at least 10% of the pseudo‐ancestors of a given person need to have two same‐ethnicity last names. We introduced this filter to maximise the probability that surnames are, indeed, representative of the Jewish and Mapuche groups. Second, we removed persons with less than two pseudo‐ancestors. Table 2 describes the datasets utilized in all eight regressions.

**TABLE 2:** Descriptive statistics for the variables in the models.

	SES	Age	Surn. size (pat.)	Surn. size (mat.)
All Jews (*N* = 16,612)				
Mean	82.31	48.77	26,968	41,252
0.25	80.03	33.00	39	40
0.50	87.80	52.00	114	153
0.75	93.47	65.00	449	2978

	SES	Endog. (mean)	Age	Surn. size (pat.)	Surn. Size (mat.)	Surn. past size (mean)	Fam. age (mean)
Two Jewish surnames (*N* = 5314)							
Mean	88.69	0.6035	55.25	127.9	144.2	46.17	1924
0.25	84.80	0.4302	37.00	24.0	24.0	10.00	1916
0.50	89.91	0.6197	62.00	55.0	55.0	22.50	1924
0.75	95.49	0.7744	69.00	135.0	151.0	52.00	1933

	SES	Endog. (pat.)	Age	Surn. size (pat.)	Surn. size (mat.)	Surn. past size (pat.)	Fam. age (pat.)
Paternal Jewish surname only (*N* = 7120)							
Mean	79.79	0.4167	46.63	183.4	95,712.0	73.23	1919
0.25	77.56	0.2000	31.00	43.0	153.8	15.00	1908
0.50	86.62	0.3711	45.00	98.0	13,147.0	35.00	1919
0.75	92.45	0.6000	62.00	227.0	117,960.0	89.00	1931

	SES	Endog. (mat.)	Age	Surn. size (pat.)	Surn. size (mat.)	Surn. past size (mat.)	Fam. age (mat.)
Maternal Jewish surname only (*N* = 5152)							
Mean	80.16	0.3776	46.01	88,309	203.7	82.03	1917
0.25	77.05	0.1667	31.00	136	44.0	18.00	1906
0.50	86.69	0.3214	44.00	6568	114.0	42.00	1917
0.75	92.86	0.5345	61.00	105,337	245.0	102.00	1929

	SES	Age	Surn. size (pat.)	Surn. size (mat.)
All Mapuches (*N* = 154,928)				
Mean	36.040	41.66	62,845	62,845
0.25	13.846	29.00	1047	1047
0.50	31.694	41.00	3459	3459
0.75	55.454	53.00	56,405	56,405

	SES	Endog. (mean)	Age	Surn. size (pat.)	Surn. size (mat.)	Surn. past size (mean)
Two Mapuche surnames (*N* = 39,955)						
Mean	34.660	0.6457	47.85	2225	2181	823.6
0.25	12.717	0.5861	37.00	639	611	262.5
0.50	29.568	0.6549	48.00	1616	1568	561.5
0.75	53.231	0.7185	60.00	3235	3152	1122.5

	SES	Endog. (pat.)	Age	Surn. size (pat.)	Surn. size (mat.)	Surn. past size (pat.)
Paternal Mapuche surname only (*N* = 53,314)						
Mean	36.470	0.5657	40.46	2221	153,556	1056
0.25	14.187	0.4912	28.00	650	29,566	243
0.50	32.226	0.5889	37.00	1601	87,357	632
0.75	55.963	0.6660	52.00	3244	221,838	1488

	SES	Endog. (mat.)	Age	Surn. size (pat.)	Surn. (mat.)	Surn. past size (mat.)
Maternal Mapuche surname only (*N* = 62,118)						
Mean	36.550	0.5585	38.74	153,370	2173	1094
0.25	14.272	0.4832	27.00	29,934	614	261
0.50	32.567	0.5823	36.00	89,031	1532	662
0.75	56.149	0.6572	49.00	221,838	3152	1528

## 6 RESULTS

### 6.1 Jewish community

The quantitative analysis substantiates the EMED's prediction that within Santiago's Jewish communities, exogamy is intricately linked with socioeconomic status (SES). Detailed in Table 3, the quantile regression outputs for the Jewish community delineate the correlation between median SES and various factors, with a particular focus on parental and ancestral endogamy across different subsets of the Jewish population.

**TABLE 3:** Quantile regressions estimating the association between endogamy and *median* SES for Jews.

	Dependent variable: Socioeconomic status rank			
	Mat. J. surname	Pat. J. surname	Two J. surnames	All Jews
Parental endogamy (two vs. one Jewish surname)				2.637*** (0.493)
Ancestral endogamy (mean)			0.598 (1.340)	
Ancestral endogamy (paternal)		4.285*** (2.251)		
Ancestral endogamy (maternal)	5.352*** (2.283)			
Gender (male)	−0.343 (0.844)	−0.227 (0.603)	0.008 (0.448)	−0.204 (0.342)
Age	−0.021 (0.031)	0.004 (0.021)	0.023*** (0.016)	0.003 (0.012)
Family age (mean)			0.010 (0.032)	
Family age (paternal)		0.026 (0.041)		
Family age (maternal)	0.003 (0.047)			
Surname size (paternal)	−0.00001*** (0.00000)	0.002* (0.002)	−0.0002 (0.001)	−0.00001*** (0.00001)
Surname size (maternal)	0.00005 (0.002)	−0.00001*** (0.00000)	−0.0001 (0.001)	−0.00001*** (0.00000)
Intercept	80.832* (91.344)	35.809 (78.321)	69.722** (61.043)	87.229*** (0.671)
Observations	5052	7120	5277	16,612

*p < 0.1; **p < 0.05; ***p < 0.01; values in parentheses represent standard errors.

The regression coefficients presented in Table 3 suggest that Jews at the lower end of the community's SES spectrum are more likely to engage in exogamy, as reflected by a marked increase in SES ranking associated with having two Jewish surnames. Specifically, the shift from one to two Jewish surnames corresponds to a 2.637‐point increase in median SES rank. This trend, however, is not mirrored for mean ancestral endogamy amongst individuals with two Jewish surnames, where the correlation with SES is positive but not statistically significant.

The impact of ancestral endogamy on SES is more pronounced among Jews with only one Jewish surname, whether paternal or maternal. Here, a more endogamous background is associated with a substantial four to five point increase in median SES rank, highlighting the significant socioeconomic uplift associated with endogamy for these individuals.

**FIGURE 9:**
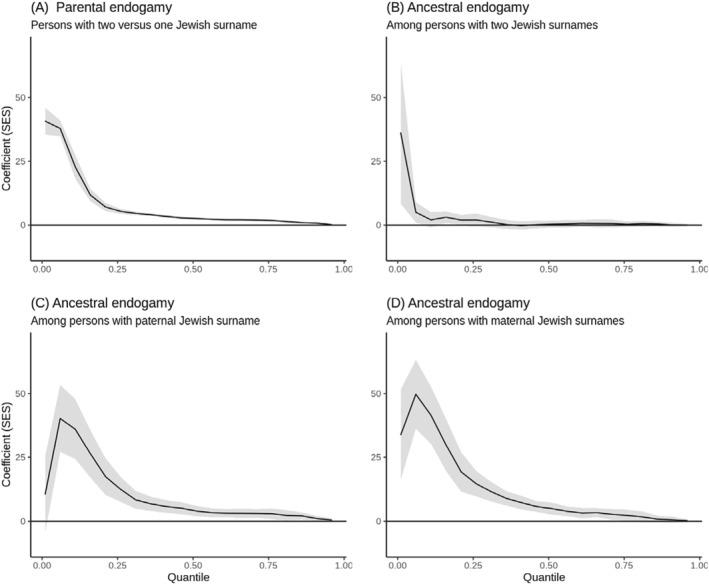
Quantile regressions coefficients establishing the association between Jewish endogamy and SES across all quantiles of the outcome variable. The shaded areas of the graphs represent confidence intervals. The quantiles calculated go between 0.01 and 0.96 in 0.05 steps. The model controls are the same as in Table 3.

Figure 9 enriches this narrative by visually representing the quantile regression coefficients and their association with Jewish endogamy across the entire range of SES outcomes. It illustrates that the socioeconomic benefits of having two Jewish parents are most evident at the lower SES quantiles. For example, at the 11th percentile, individuals with two Jewish surnames are situated 22 rank‐points higher than those with only one Jewish surname. Yet, at the 91st percentile, the advantage narrows to a mere 0.7 rank‐points, emphasizing that the benefits of endogamy are more acute for those at the lower end of the SES scale.

In the case of ancestral endogamy, the effects are nuanced. For individuals with two Jewish surnames, significant increases in SES rank due to ancestral endogamy are limited to the lowermost deciles of the SES distribution. This contrast is stark for those with a single Jewish surname, as shown in Figure 9, where the impact of ancestral endogamy on SES rank is both pronounced and statistically significant across a broader spectrum of SES quantiles, especially for those with a maternal Jewish surname.

These findings, particularly for the Jewish community, affirm the hypothesis that socioeconomic outliers within ethnic groups—those with atypical SES—are more likely to marry out and have children who do not retain the ethnic identity. This pattern not only contributes to the socioeconomic homogenization of the group over time but also reinforces the community's prevailing economic profile. The empirical evidence suggests that the Jewish community's relatively high SES in Chile is partly self‐perpetuating due to the outmigration of its poorer members, while the Mapuche lower SES is maintained due to the exodus of its wealthier individuals.

### 6.2 Mapuche community

The analysis of the Santiago Mapuche, as seen in Table 4, contrasts with that of the Jewish community, indicating a distinct socioeconomic pattern. The Mapuche, generally less affluent and predominantly in the lower third of the city's SES distribution, exhibit a negative correlation between endogamy and SES, a reversal of the pattern observed among Jews.

**TABLE 4:** Quantile regression coefficients predicting *median* SES from endogamy at the parental and ancestral levels for Mapuches.

	Dependent variable: Socioeconomic status			
	Mat. M. surname	Pat. M. surname	Two M. surnames	All Mapuches
Parental endogamy (two vs. one Mapuche last name)				−2.673*** (0.619)
Ancestral endogamy (mean)			−9.112*** (4.822)	
Ancestral endogamy (paternal)		−15.908*** (4.068)		
Ancestral endogamy (maternal)	−16.233*** (4.485)			
Gender (male)	−1.258*** (0.812)	−1.507*** (0.759)	−4.459*** (0.817)	−2.275*** (0.419)
Age	0.002 (0.033)	−0.003 (0.029)	−0.130*** (0.032)	−0.066*** (0.015)
Surname size (paternal)	−0.00000 (0.00000)	−0.00004 (0.0003)	−0.0002** (0.0002)	−0.00000 (0.00000)
Surname size (maternal)	0.0001 (0.0003)	−0.00000 (0.00000)	−0.0001 (0.0002)	−0.00000 (0.00000)
Intercept	42.295*** (2.715)	42.511*** (2.546)	44.456*** (3.095)	36.427*** (0.796)
Observations	62,118	53,314	39,955	154,928

*p < 0.1; **p < 0.05; ***p < 0.01; values in parentheses represent standard errors.

Table 4's quantile regression results highlight a consistent negative association between both parental and ancestral endogamy and median SES for Mapuches. Parental endogamy results in a 2.673‐point median decrease in SES for individuals with two Mapuche surnames compared to those with one. Ancestral endogamy, particularly for those with one Mapuche surname—paternal or maternal—shows an even larger negative impact, with a 15.908–16.233‐point median decrease in SES, indicating that Mapuches with a more endogamous background tend to be lower in SES than their counterparts from more mixed backgrounds.

**FIGURE 10:**
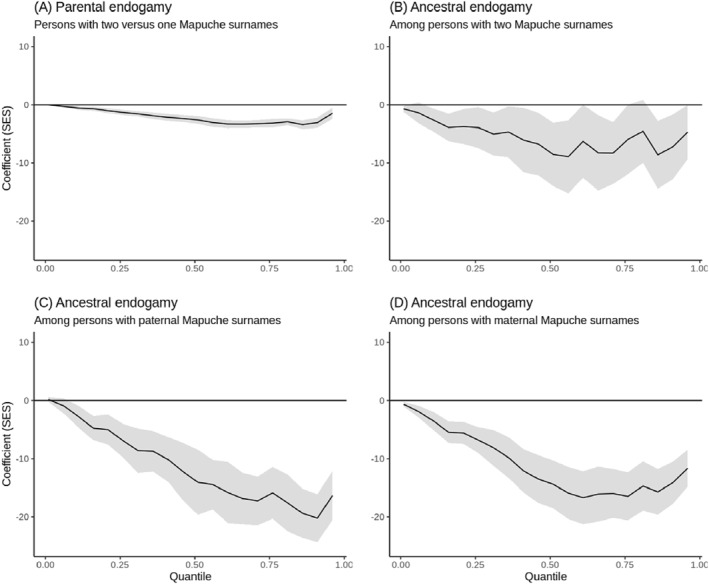
Quantile regressions coefficients establishing the association between Mapuche endogamy and SES across all quantiles of the outcome variable. The shaded areas of the graphs represent confidence intervals. The quantiles calculated go between 0.01 and 0.96 in 0.05 steps. The model controls are the same as in Table 4.

Figure 10 further delineates this relationship across the SES distribution, where the negative association between endogamy and SES is persistent across all subgroups. Unlike the pattern for Jews, where endogamy's positive effect on SES was most pronounced at lower SES levels, for Mapuches, the negative impact of endogamy is more significant in the middle and upper SES brackets. This suggests that for Mapuches, the socioeconomic disadvantages of parental and ancestral endogamy are more impactful among those who are better off.

For instance, Figure 10A shows that at the sixth SES percentile, Mapuches with one surname are marginally higher in rank than those with two Mapuche surnames. Conversely, at the 91st percentile, the difference widens substantially, with those having one Mapuche surname being 3.1 rank‐points higher than their counterparts with two Mapuche surnames. This pattern indicates that the negative socioeconomic impact of endogamy among the Mapuche is accentuated for those at the higher end of the SES spectrum.

In summary, the quantile regressions elucidate a clear socioeconomic distinction within Mapuches: more endogamous individuals tend to occupy the lower rungs of the socioeconomic ladder, whereas those with mixed backgrounds are relatively better off. This inverse relationship between endogamy and SES among Mapuches is a significant finding, contrasting sharply with the positive correlation observed among the Jewish community of Santiago.

## 7 DISCUSSION AND CONCLUSION

Our empirical analysis confirms that within the Jewish and Mapuche groups of Santiago, exogamy correlates with socioeconomic status. Specifically, Jewish individuals at the lower socioeconomic tier and Mapuches at the higher tier are more likely to have ethnically mixed parentage. Since parent‐child incomes are highly correlated in highly unequal societies like Chile's (UNDP, 2017), these findings suggest that exogamous marriages are significantly more likely to involve ethnic group members who are socioeconomically atypical relative to the rest of their group, who in turn pass their atypical socioeconomic status to their mixed‐ethnic children.

Our findings challenge the conventional sociological construal of exogamy as a characteristic of more educated ethnic minority members and an indicator of migrant assimilation (Blackwell & Lichter, 2004). Contrasting with this assumption, the finding that Jews in higher socioeconomic positions are less likely to intermarry reveals that a more significant predictor of exogamy is the socioeconomic position of individuals relative to the rest of their ethnic group. This interpretation is consistent with the findings of studies of highly‐educated American ethnic and migrant minorities, namely Asian‐Americans (e.g., Qian & Qian, 2020) and American‐Jews (DellaPergola, 2003; Philips & Fishman, 2006). More to the point, Kalmijn's (2012) statistical analysis of 46 immigrant groups found an “educational gradient” in minorities spousal choices, such that “[i]n lower‐educated immigrant groups, the higher educated will have fewer chances of meeting a coethnic spouse than the lower educated […] In higher‐educated immigrant groups, in contrast, higher educated members will have better chances to meet a coethnic spouse of the same level of education than the lower educated” (Kalmijn, 2012, p. 456).

Our findings support and extend Kalmijn's “educational gradient” thesis. We find that a “gradient” can also characterize the relationship between exogamy and income, not just education. Moreover, this relationship is shown to exist outside the US context, and in connection with indigenous (Mapuches) and long‐established minority groups. In one critical aspect, however, our findings challenge Kalmijn's thesis and similar findings obtained in the wider literature on ethnicity and marriages: we find strong evidence for the existence of a negative status “gradient” when it comes to Jews' marriage patterns.

Our findings also suggest that observed gradations in endogamy patterns function to consolidate the socioeconomic “niches” of the Jewish and Mapuche communities within Santiago's larger population over time. EMED represents a complementary model to traditional sociological explanatory models of ethnic minority economic outcomes. It suggests that higher intermarriage rates among socioeconomic outliers may reflect the latter's prior structural sorting into interactional settings with fewer co‐ethnic marital candidates, or the stronger attraction felt by individuals towards those with similar socioeconomic backgrounds and statuses (Skvoretz, 2013).

While this study focused on two distinct ethnic groups, EMED could be applicable to any distinct social category with distinguishable “typical” and “atypical” socioeconomic members. Future research should test whether our model holds for ethnic groups having different geographies and ethnoreligious customs, potentially refining or challenging the EMED framework presented here. This study also highlighted the potential influence that ancestral endogamy wields on individuals' socioeconomic statuses, encouraging future studies to examine individual economic outcomes as a function of wider kin network attributes and multi‐generational processes extending beyond the parental generation.

In closing, it is worth noting that the cross‐sectional design of our dataset limits our ability to determine the causal direction between socioeconomic status and marriage. Future works could improve upon this by employing longitudinal data to better understand the dynamics at play, particularly how socioeconomic status may influence or be influenced by marital choices across generations.

## CONFLICT OF INTEREST STATEMENT

None of the authors have a conflict of interest to disclose.

## Figures and Tables

**FIGURE A1:**
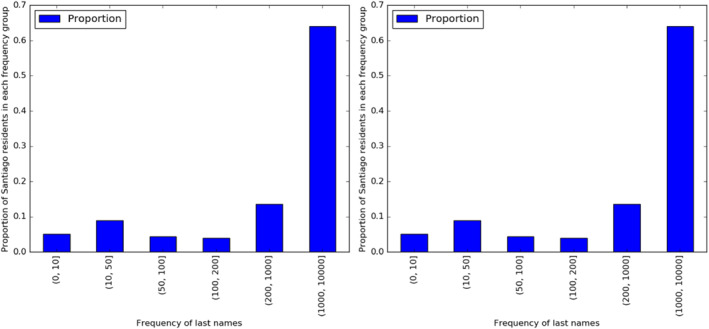
Frequency of all surnames.

## Data Availability

The data that support the findings of this study are available from the corresponding author upon reasonable request.

## ENDNOTES

^1^
We use the term “community” to refer to the respective group members living in Santiago; we acknowledge that other terms, such as “people,” can be used to refer to these groups more globally.

^2^
This list was accessed on October 19, 2020.

^3^
These lists have been made available in digital format by the Mapuche Data Project at http://mapuchedataproject.cl/.

^4^
The last names were Con, Neiman, Neuman, Levin, Levi, and Rein.

^5^
We have access to the electoral registry of 2021, which contains a column indicating whether voters belong to a recognized native Chilean group. These data allows us to assess how useful is the surname‐based strategy to identify the Mapuches of Santiago. The registry contains 4,139,863 names, of which 205,637 are Mapuche. Using surnames, 159,872 individuals hold at least one Mapuche surname, of which 92% are registered as Mapuche in the official records.

^6^
This dataset was provided directly by the Servicio de Registro Civil e Identificación de Chile after we applied for it following Chile's so‐called “law of transparency”.

^7^
Kalmijn's formula for calculating endogamy within an ethnic group C is (C_AA_/C_AB_)/(C_BA_/C_BB_), where C_AA_ is the amount of co‐ethnic marriages within the group, C_AB_ is the amount of mixed marriages involving a co‐ethnic male and a female outsider, C_BA_ is the amount of mixed marriage involving a female co‐ethnic and a male outsider, and C_BB_ is the amount of marriages involving outsiders only.

^8^
We thank Andrés Cruz and Josué Tapia for helping with geocoding.

^9^

https://github.com/fvillena/rut‐a‐edad.

## APPENDIX 1

Most residents of Santiago have surnames that are held by more than 1000 people, as Figure A1 shows. This distribution differs from that of Jews and Mapuches, that tend to hold rare last names.
